# Current Therapeutics and Future Perspectives to Ocular Melanocytic Neoplasms in Dogs and Cats

**DOI:** 10.3390/bioengineering8120225

**Published:** 2021-12-20

**Authors:** Tarcísio Guerra Guimarães, Karla Menezes Cardoso, Pedro Tralhão, Carlos Miguel Marto, Nuno Alexandre, Maria Filomena Botelho, Mafalda Laranjo

**Affiliations:** 1 Institute of Research and Advanced Training (IIFA), University of Évora, 7002-554 Évora, Portugal; tarcisioguerra77@gmail.com (T.G.G.); cardosokarla86@gmail.com (K.M.C.); 2 Mediterranean Institute for Agriculture, Environment and Development (MED), University of Évora, 7006-554 Évora, Portugal; nmla@uevora.pt; 3 Institute of Biophysics, Faculty of Medicine, University of Coimbra, 3000-548 Coimbra, Portugal; cmiguel.marto@uc.pt (C.M.M.); mfbotelho@fmed.uc.pt (M.F.B.); 4 Institute for Clinical and Biomedical Research (iCBR), Area of Environment, Genetics and Oncobiology (CIMAGO), Faculty of Medicine, University of Coimbra, 3000-548 Coimbra, Portugal; 5 Center for Innovative Biomedicine and Biotechnology (CIBB), University of Coimbra, 3000-548 Coimbra, Portugal; 6 Center of Veterinary Ophthalmology, Oftalvet, 4050-102 Porto, Portugal; pt@oftalvet.pt; 7 Institute of Experimental Pathology, Faculty of Medicine, University of Coimbra, 3000-548 Coimbra, Portugal; 8 Clinical Academic Center of Coimbra (CACC), 3004-561 Coimbra, Portugal; 9 Department of Veterinary Medicine, University of Évora, 7004-516 Évora, Portugal

**Keywords:** melanocytic neoplasms, melanoma, therapeutics

## Abstract

Neoplasms of melanocytic origin are diseases relevant to dogs and cats’ ophthalmic oncology due to their incidence, potential visual loss, and consequent decrease in life quality and expectancy. Despite its non-specific clinical presentation, melanocytic neoplasms can be histologically distinguished in melanocytomas, which present benign characteristics, and malignant melanomas. The diagnosis often occurs in advanced cases, limiting the therapeutic options. Surgery, cryotherapy, radiotherapy, photodynamic therapy (PDT), and laser are currently available therapeutic strategies. As no clinical guidelines are available, the treatment choice is primarily based on the clinician’s preference, proficiency, and the owner’s financial constraints. While surgery is curative in benign lesions, ocular melanomas present a variable response to treatments, besides the potential of tumour recurrences or metastatic disease. This review presents the currently available therapies for ocular melanocytic neoplasms in dogs and cats, describing the therapeutic, indications, and limitations. Additionally, new therapeutics being developed are presented and discussed, as they can improve the current treatment options.

## 1. Introduction

Veterinary ophthalmologic diseases comprise a wide range of diseases affecting the orbit, globe, and adnexa structures. About 39% of ocular pathological processes in small animals correspond to neoplasias [1], the abnormal growth of a tissue into a mass that is not responsive to homeostatic control mechanisms and may be benign or malignant [2]. However, due to its location and unique eye anatomy and function, even small, non-invasive, and slow-growing neoplasms can cause significant discomfort, colour changes, alterations in the eye bulb shape, and vision loss [3,4,5]. 

Melanocytic neoplasms are the most common form of primary intraocular neoplasia, and, among those, melanomas are highly prevalent, with different incidences regarding species and anatomical location. Melanomas are histologically confirmed tumours with malignant characteristics, and melanocytomas are benign tumours with no metastatic potential [6,7,8]. Melanomas originate from melanocytes or other neoplastic cells that develop from melanocytes or melanoblasts [9]. Although benign, melanocytomas have the capacity for local expansion and impairment of adjacent structures [5,10]. Additionally, variations in the biological behaviour and malignancy exist, according to the lesion location and animal species. The growth rate of neoplasms of melanocytic origin is variable [4]. In addition, there is a lack of reports with adequate follow-up information to determine if the histological classification accurately indicates the neoplasm behaviour [11].

**Figure 1:**
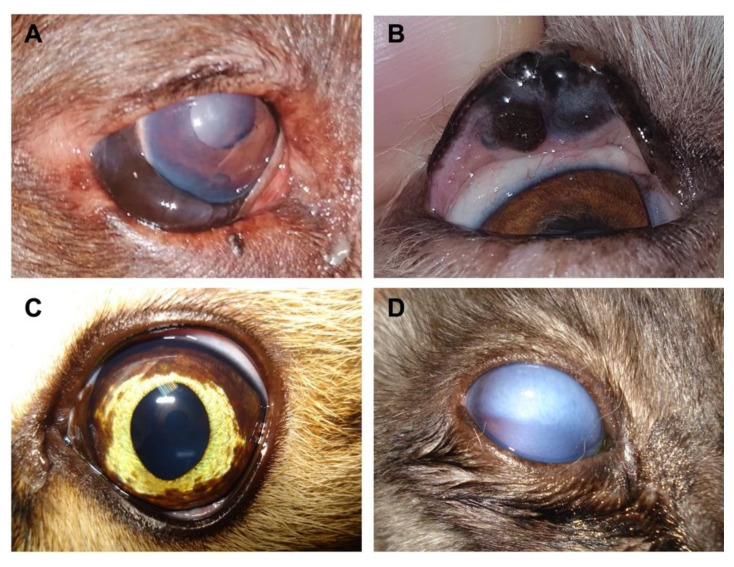
Clinical presentation of several ocular melanocytic tumours diagnosed in dog and cat patients: (**A**) a dog with expansive/invasive neoformation in the epibulbar region, which is intensely pigmented and with the presence of corneal oedema; (**B**) a dog with neoformation affecting the superior eyelid in a large extension; (**C**) a cat with a left eye showing an alteration of pigmentation, which may reflect a manifestation of diffuse melanoma; (**D**) a cat with a right eye affected with glaucoma secondary to a diffuse neoformation.

Melanocytic neoplasms clinical presentation is non-specific and varies according to the eventual destruction or disarrangement of ocular structures [12]. Unilateral involvement is the most frequent [9]. Clinical signs include visual loss, corneal opacity, hyphema, endophthalmitis, uveitis, retinal detachment, presence of expansive/invasive neoformations [12,13] (see Figure 1A,B), alterations of pigmentation (see Figure 1C) and secondary glaucoma (common; see Figure 1D) [14]. The presence of secondary glaucoma is an indicator of poor prognosis and reduced survival time in cats with diffuse iris melanoma [15]. Moreover, specific histological and immunohistochemical characteristics relate to poor diagnosis, as further discussed [15,16,17]. Besides pigment deposition (hyperpigmentation) [18,19], also present in other neoplasms such as basal cell tumours [20], melanomas may also present a non-pigmented form, named amelanotic [5,13,21,22]. 

Melanocytic neoplasms tend to occur in adult to elderly animals [13,23], but there are cases in young animals [24]. Gender [13,25] or breed predisposition is yet to be confirmed [7,20,23,26,27,28], since the apparent higher prevalence in some breeds may be related to their popularity and not predisposition [9].

The aetiology of ocular melanoma is not fully elucidated [9], but some factors may be related, such as genetic [29], pigmented region lesions [2,15,30], exposure to UV radiation [31] and possible viral infection [32]. Experimental studies showed uveal melanomas could be induced in dogs with the intravenous administration of radioactive material [33]. Additionally, intraocular injection of feline sarcoma virus in neonatal kittens could induce the formation of invasive anterior uveal melanoma [34]. These findings relate cell damage to lesion appearance; although, as referred, the aetiology is not yet fully elucidated. As melanoma, melanocytoma often originates in pigmented eyes or those presenting melanosis [1,35,36], which can be a hereditary condition or consequent to an inflammatory process [35,37,38]. 

In dogs, the melanocytic neoplasms reported in the conjunctiva [10,39] and the orbit [2,20] are predominantly malignant, while those originating in the eyelid (see Figure 1D), the limbus and the uvea are predominantly benign [1,2,5,6,20,29,40]. Melanomas with areas of melanocytoma were also observed, suggesting malignant transformation [9]. Cats are often diagnosed with intraocular melanocytic neoplasms, which diffuse in the iris [41] and gradually become a coalescing infiltrative lesion [14]. These lesions have variable progression rates and present histological and clinical changes during the neoplasm evolution. Diverse histological characteristics can be observed within the neoplasm [42], such as atypia, mitotic index, or nuclear/cytoplasm ratio [11]. Other than iris, uncommon localisations include the ciliary body, the limbus [43,44,45], the optic nerve, the iridocorneal angle, the sclera, the episcleral [43], the conjunctiva [27,46,47,48], the choroid [14,43,49], the nictitating membrane [50], the eyelid [51,52], and the orbit [46,47,53].

In most dogs and cats, intraocular melanomas are diagnosed at advanced stages, limiting the treatment eligibility and the prognosis [12]. When evaluating a possible ocular neoplasm, a detailed anamnesis and a general physical examination should be performed [54]. In addition, a complete eye examination should be carried out [20], including slit-lamp biomicroscope magnification, ophthalmoscopy, tonometry, ocular ultrasound, and gonioscopy [12,20,55]. When required, magnetic resonance imaging and computed tomography can be performed, providing a detailed and complete assessment of the orbital region [20,38,56]. The complementary diagnostic exams are essential to evaluate the involved structures, identify the neoplasia’s margins and extent, and define the most appropriate treatment strategy [25]. 

Ocular neoplasms may be of primary origin, arising from the affected eye tissue itself, or secondary, originating from a site distant from the ophthalmic region [10,13,57]. Eye melanomas of metastatic origin generally have oral and nail bed melanomas at the primary site [5,9,13,58].

Eye melanomas most common metastasising route is the haematogenous dissemination through conjunctival or periorbital vessels [2,4]. Other possibilities are the optic nerve and local invasion through adjacent tissues, such as the sclera and the cornea [2,4]. In dogs, metastases may occur according to the site of neoplasm origin, the extension of ocular infiltration and mitotic index [7,59,60,61]; affecting structures such as lymph nodes [39,62], liver [57], lungs [63], brain [64], vertebra [65], kidneys and prostate [62]. In cats, ocular melanoma shows a metastasising preference for distant organs such as the liver, the lungs [17,47] and the kidneys [5], in addition to unusual locations such as the radius head [66]. A case involving multiple locations (lungs, pericardium, parietal pleura, mediastinum, hilar lymph nodes, diaphragm, liver, and omentum) was reported [67].

**Figure 2:**
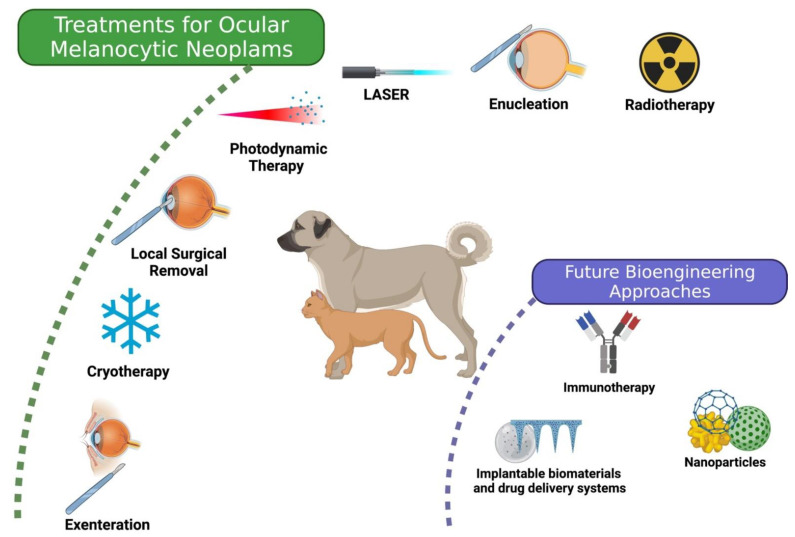
Therapeutic strategies currently available for melanocytic neoplasms. Created with BioRender.com (accessed on 15 December 2021).

Several therapeutic strategies are currently available for melanocytic neoplasms: surgery, cryotherapy, radiotherapy, photodynamic therapy (PDT), and laser. Additionally, new treatments are being developed, as schematized in Figure 2.

The treatment indication should consider general health status, species, the biological behaviour of melanocytic neoplasms, lesion characteristic, lesion progression, anatomical site of the lesion, lesion extension, visual or blind eye, presence of secondary ocular changes, clinician preference and proficiency, and owner’s financial constraints.

Image documentation (photography) can be used to monitor any changes in pigmented eye lesions prior to treatment indication. Because infrared light is less susceptible to scattering, infrared photography is an ideal method for identifying small, often indiscernible areas of hyperpigmentation of the iris, even in heavily pigmented eyes or even when a cloudy or opaque cornea is present [38]. Both strategies allow tracking the progression of eye lesions [38,68]. The surgical approach is usually curative in cases of melanocytoma [49,69,70,71]. Ocular melanomas present variable response rates to treatments [38]. Despite the different available options, there is a lack of clinical evidence systematisation to support the therapeutic decision. As with any form of ocular cancer, preoperative chest X-rays and abdominal ultrasonography for metastasis detection are recommended [4,54]. For anatomopathological study and lesion staging, samples can be obtained by excisional, incisional, or fine needle aspirate, depending on the neoplasm location [42,72,73]. Confirmation of melanocytic origin can be performed with immunostaining [17,20]. 

The variable response rates, the visual loss and the impact on the patient’s life expectancy make the development of new therapies extremely relevant [10,12,74]. Therefore, this manuscript aimed to review the currently available and under research treatments for ocular melanocytic neoplasia in dogs and cats.

## 2. Treatments

The National Comprehensive Cancer Network (NCCN) Guidelines provide a clear guide to ocular melanoma therapeutic options, according to tumour size [75]. Treatment can be either enucleation or globe preserving [76]. For most tumours’ radiotherapy is an option. Nevertheless, several effective options are now approved for adjuvant therapy besides observation, including immunotherapies [75]. However, in Veterinary Medicine, no clinical guidelines are available for the treatment of eye neoplasms. Decisions are made primarily based on the clinician preference, proficiency [77], and the owner’s financial constraints [38]. In addition, the indication should consider each patient’s health, aiming to preserve the affected eye vision [78] and avoid metastatic dissemination [7,57]. The knowledge of melanocytic neoplasms biological behaviour and characteristics, the affected area’s progression, the affected structures and the patient’s species are essential in therapeutic planning [20,38,79].

### 2.1. Local Surgical Removal

Local surgical excision can be performed when there is no invasion or compromise of adjacent structures, and the neoplasm site and extent allow total removal, with generous free margins, but with eye globe preservation [20,38]. Co-adjuvant treatments may be employed after surgical excision [20,42,52,80,81]. Patients should be closely monitored in follow-up consultations, with clinical evaluation of the ocular structures [77]. Particular attention must be given to complications related to glandular portion removal [82,83,84].

Wide excision may be required when the eyelid region is affected [38]; although, the species characteristics must be considered. For example, most lid neoplasms in cats are malignant and wider excision is necessary, often requiring blepharoplasty, sliding, and eyelid grafting of adjacent or facial tissue [85,86]. 

Small isolated conjunctival melanomas which not compromise the adjacent structures may undergo complete surgical resection [38]; however, sometimes conjunctival transposition may be required [20,86]. In addition, the third eyelid may undergo partial excision or total removal [20,86].

In the iris, small, isolated neoplasms with well-defined borders can be submitted to local excision with iridectomy (a section of total iris thickness is extirpated) or iridociclectomy (a portion of the iris and adjacent ciliary body is excised), which may include removal of the adjacent sclera. In cats, minor complications have been reported as mild intra-operative haemorrhage, fibrin clot, corneal ulcer, postoperative ocular hypertension, dyscoria, and pseudopolycoria [37]. Sclerectomy, with or without lamellar keratectomy, may be used as a treatment for canine and feline limbal melanomas that exhibit benign biological behaviour [29,44].

The sectoral exeresis techniques of isolated neoplasms may be successful. However, if the neoplasm invades the sclera or ciliary body, the success may be limited, with the risk of postoperative complications resulting in the need for ocular bulb excision or *phthisis bulbi* [86]. Still, as a form of repair and to preserve the anatomy and visual function, different grafts can be applied after the surgical removal of ocular melanoma [45,70,78,81,87,88].

### 2.2. Laser

Different lasers are used to treat various conditions and ophthalmic diseases [72,89,90] such as iris cyst, capsular opacification, glaucoma, retinal detachments, and pigmented ocular neoplasms [72,91]. Depending on the wavelength, intensity, and duration of exposure, tissues can absorb energy, so the laser can be adjusted to perform photocoagulation, thermotherapy, and photodynamic therapy or undergo ionisation with photodisruption action [92]. The most commonly used lasers in veterinary ophthalmology are neodymium:yttrium-aluminium-garnet (Nd:YAG), diode and CO_2_ [89]. The laser’s effect depends on the absorption by eye pigments, so the grade and type of eye pigment (melanin, haemoglobin and xanthophyll) and its absorption play important roles in phototherapy [93]. Diode lasers are especially attractive to veterinary ophthalmologists because of their high melanin absorption, which is particularly useful in veterinary patients’ typically heavily pigmented eyes [89]. 

Nd:YAG laser is used to treat limbal melanoma in dogs and cats, reducing pigmented tissues after treatment [70,88,94]. Diode laser photocoagulation has demonstrated excellent long-term results, is technically easy to perform, minimally invasive, and well-tolerated [95], in addition to its preferential absorption in tissues containing melanin, such as melanomas [72,96]. Other advantages of laser are avoiding intraocular complications, requiring only a short duration of general anaesthesia, and not requiring donor graft tissues. However, its main limitation is the higher recurrence rate [38].

Neoplasms with flat and small dimensions, such as isolated iris melanoma, can be considered for laser photocoagulation if successful laser penetration is predicted [72,96]. Dogs with isolated iris melanomas were treated using a laser diode through an operative microscopic adapter or an indirect ophthalmoscope. An immediate decrease in masses was observed. In neoplasms with a mean thickness greater than 2 mm, additional laser therapy may be required [72,96]. The energy adjustment should be made carefully to avoid dispersion of pigmented tissue and iatrogenic damages [72,96]. Secondary alterations after treatment have been reported, as focal corneal oedema, dyscoria [90], uveitis, hyphema, and cataract [88]. However, as previously stated, it is considered a minimally invasive and well-tolerated technique compared with other therapeutic modalities [88].

In cats, it is possible to ablate small, isolated, and hyperpigmented foci of the iris [97] through a diode laser photocoagulation, which induces lesion contraction [98]. However, due to the multifocal nature of these neoplasms, new lesions may arise [99]. Moreover, without previous histological examination, there is the risk of spreading cells and, consequently, metastases [20,98]. Therefore, although the use of laser on felines pigmented lesions has been suggested, it remains controversial [38]. In contrast to cats, anterior uveal melanomas in dogs metastasise late and less frequently. Therefore, laser photocoagulation is a more attractive approach to eliminating the lesion while preserving eye structure and vision [38].

For anterior uveal neoplasms, newer endoscopic diode lasers may prove to be even more beneficial. This technique uses a 20 g endoprobe that provides light, video, and diode laser. This technique allows accessing the posterior iris and ciliary body to precise visualisation and delivery of laser energy while maintaining the anterior chamber structure and avoiding eye collapse [38].

Isolated neoplasms on the third eyelid can benefit from CO_2_ laser excision, which proved efficient and effective, with excellent postoperative comfort and minimal complications [84]. However, when neoplasms are extracted using heat-generating devices, caution should be taken due to the samples impairment, which can be difficult for the histological analysis [100]. 

Additionally, due to their highly coherent monochromatic light emission, lasers are used as light delivery devices in photodynamic therapy, as described below.

### 2.3. Photodynamic Therapy

Photodynamic therapy is an evolving modality for treating various diseases, including ocular neoplasms. Its therapeutic effect involves photochemical reactions mediated by the interaction of photosensitising agents, light, and oxygen [101,102,103], which culminates in the oxidation of cancer cells and leads to apoptosis, necrosis, antiangiogenic effects, and immune stimulation [102,103]. PDT is minimally invasive, relatively non-toxic and has no carcinogenic or mutagenic effects [101]. Side effects of photodynamic therapy in veterinary medicine are rarely observed [104,105]. However, pruritus, photosensitisation [104], and increased body temperature [101] were occasionally reported [106].

Although several reports exist on PDT use in veterinary medicine, this treatment is not part of the standard treatments for ocular neoplasms [107,108,109,110,111,112,113]. However, in human medicine, PDT is indicated in treating several eye conditions, which justifies its interest in treating veterinary eye diseases [108].

A dog with scleral melanoma was submitted to photodynamic therapy with the photosensitiser 5-ethylamino-9-diethylaminobenzo[a]phenothiazine chloride (EtNBS), administered intravenously (2.0 mg/kg), and irradiated with a diode laser until 200 J/cm^2^. Although a visible part of the neoplasms became paler, the neoplasm diameter remained stable two months after PDT. Corneal and local conjunctival reaction (mild) was observed consisting of chemosis and hyperaemia (grade 1) [101].

PDT was also performed in a cat with a history of four surgical resections with recurrences of eyelid melanoma. After new lesion excision, free margins were not obtained, and intraoperative PDT was performed with Acridine orange (OA-PDT) to control residual tumour cells. A xenon light of 400–700 nm wavelength and 20.7 mW/cm^2^ was applied for 10 min at 10 cm from the field. No postoperative complications were observed after treatment. After 9 months, local recurrence was detected, and new surgical resection with PDT was performed. The animal died 16 months after treatment, and at this time point, no tumour recurrence was detected on the original site. Notably, during the follow-ups, no adverse effect from PDT was detected on the surgical site [52].

However, these are the only studies where PDT usefulness was accessed in veterinary ocular melanoma. Although the limited reports in dogs and cats, PDT successfully treated intraocular melanomas induced in rabbits [114,115,116,117,118], with neoplasm growth arrest [115], irreversible regression [117], and neoplasm destruction [114,116]. In a recent systematic review comparing the available treatments for induced ocular melanomas in rabbits, all options were effective in treating ocular melanoma, with more conservative options such as photodynamic therapy presenting great potential and fewer side effects [119].

### 2.4. Cryotherapy

Cryotherapy consists of a rapid freezing phase followed by a slow thaw phase, allowing temperatures that originate the formation of intracellular crystals, which will cause cellular disruption [81]. Two cycles are generally considered sufficient to achieve cell disruption [81]. Cryogenic therapy is often used as adjunctive therapy for various ocular pathologies, including surface and benign and malignant intraocular lesions [120]. 

Several cryogenic agents are used in veterinary medicine [87,121], with different minimum temperature ranges (liquid nitrogen up to −196 °C; nitrous oxide up to −80 °C; fluoroethane up to −70 °C) [42].

Tetrafluoroethane was used in dogs with limbal melanoma [81]. The treatment consisted of lamellar resection, cryotherapy, and adjuvant graft [81]. Clinical follow-up ranged from 6 months to 8.5 years, without recurrences detection. However, some post-procedure complications have been reported as uveitis, corneal ulceration, corneal oedema, granulation tissue, dyscoria, and corneal lipidosis [81]. In addition, post-procedure inflammation may be substantial and acute. Thus, careful follow-up evaluations of all eye structures should be performed when using cryotherapy, mainly when intraocular tissues are targeted [42].

Eyelids with flat pigmented lesions or after partial surgical removal may be submitted to cryotherapy procedures [20]. However, cryotherapy is not typically curative when used to treat palpebral melanoma [85], and cutaneous depigmentation may occur after cryotherapy due to the dermal melanocytes sensitivity [122].

### 2.5. Radiotherapy

Radiotherapy involves the use of ionising radiation in the form of particles or electromagnetic waves for disease treatment, mainly of neoplastic nature [123]. Radiation therapy is the main conservative therapy for ocular melanoma in ophthalmology [124]. In veterinary medicine, radiotherapy can be performed on patients as a curative or palliative approach [125].

A retrospective study evaluated the ocular side effects of cancer-bearing dogs and cats treated with Cobalt-60 (^60^Co) external beam radiation in which one or both orbit(s) were included in the radiation field. Eyelid lesions, conjunctivitis, keratoconjunctivitis sicca, keratitis, ulcerative keratitis, cataract, uveitis, and retinal lesions as ocular side effects were reported [125].

Thirty dogs with limbal melanoma were treated with lamellar resection and adjunct radiotherapy using strontium-90 (^90^Sr) plesiotherapy [80]. With this modality of brachytherapy, a low recurrence rate was reported. Post-procedure complications included corneal scarring, corneal neovascularisation, conjunctivitis, lipid keratopathy and focal bullous keratopathy, deep scleral tapering, focal scleromalacia, globular perforation, focal bullous keratopathy, and sectoral cortical cataract [29].

This treatment method is technically simple to perform, minimally invasive, well tolerated, and highly effective but is associated with a reasonably high rate of complications [81], requiring regular ophthalmologic follow-ups [125]. Moreover, radiotherapy treatment tends to be limited due to safety precautions, licensing, and expenses related to this therapeutic modality [42].

### 2.6. Enucleation

The enucleation comprises removing the globe eye, nictitating membrane, conjunctival sac, and lid margins [38]. This surgical technique is necessary when there is no possibility of local therapy [86], by the presence of inherent and intractable secondary intraocular alterations [97], particularly in those eyes that develop secondary glaucoma [10]. Other indications for enucleations are blind and painful eyes and the assumption of species-related neoplasm aggressiveness [14,126]. Visual and comfortable eyes may be preserved, mainly when the decision is based on assessing neoplasm malignancy by incisional biopsies or cytology of intraocular aspirates [73].

In dogs, neoplasm extension, neoplasm size, and mitotic index were not reliable predictors of survival after enucleation [7]. However, in cats, the extent of diffuse iris melanoma at the time of enucleation was correlated with survival, with larger neoplasm being associated with lower survival rates [15]. In addition, extrascleral extension, necrosis within the neoplasm, a mitotic index, choroidal invasion, and increased immunostaining intensity were associated with increased metastasis in the cat after enucleation [17].

Diffuse iris melanoma cannot be clinically differentiated from benign pigmentation. Therefore, these conditions remain a dilemma for the veterinary ophthalmologist when deciding whether to excise an eye that may not be affected by a malignant process or continue monitoring an affected eye, risking metastization if it is a malignant lesion [15,28]. In addition, the enucleation procedure is associated with complications such as oedema, haemorrhage and infections [86].

### 2.7. Exenteration

Exenteration removes all orbital tissue, including the eye bulb, conjunctiva, nictitating membrane, lacrimal gland, zygomatic salivary gland, extraocular muscles, palpebral margins, and even a part of the orbital periosteum, when necessary [86]. Therefore, exenteration is a more radical therapeutic option, aiming to remove the lesion with extensive surgical margins, restricting neoplasm development and eventual recurrences [127].

This way, orbital exenteration is indicated to treat advanced stages of invasive conjunctival [27], orbital [53] melanomas and when it shows evidence of crossing the sclera [3,128]. When orbital neoplasms invade the surrounding structures, they may also require orbitectomy [86], plus adjuvant therapies [53]. Additionally, neoplasms that affect and develop in the sclera may have a poor prognosis due to the possibility of haematogenous dissemination [9].

Both enucleation and exenteration are treatments with good results in patients with ocular melanoma [42]. The complications associated with exenteration are similar to those associated with enucleation. The postoperative swelling and the long-term wound contracture are usually higher because of the more significant amounts of orbital tissues excised [86].

## 3. Future Bioengineering Approaches

Despite the several therapeutic options currently available in veterinary practice, the treatment effectiveness and eye globe and vision conservation still need to be improved. 

Research in veterinary medicine points to new perspectives in the treatment of this pathology. For instance, while there is no evidence that chemotherapy is effective against melanoma [9,129], studies on the KIT proto-oncogene receptor tyrosine kinase (*KIT*) in feline iris melanoma gene expression suggest a potential chemotherapy target [41,130] that needs to be explored. 

A xenogeneic human tyrosinase DNA vaccine was bioengineered for the treatment of oral malignant melanoma, being recommended after local control has been achieved [20,131,132]. The experimental vaccine was administrated in a clinical study on melanoma patients, including a dog with ocular presentation (previously treated with exenteration). While this approach still needs optimisation, some patients showed long-term survivals [132]. Following this study, the vaccine was evaluated in 24 cats with melanoma. Four of these animals had ocular/periorbital topography affected. Despite some adverse effects (pigmentation at the application site, nausea, depression, inappetence, muscle fasciculations, and pain administration), the safety of the vaccine was established. However, efficacy and survival were not reported [131].

Further studies show encouraging potential to treat ocular melanomas in humans and animals. Regarding these, the use of implantable biomaterials and drug delivery systems present as therapeutic advancements for various ocular diseases [133]. 

The treatment of melanoma was demonstrated in a murine model, using bioresorbable, miniaturised porous silicon (p-Si) needles with covalently-linked drug cargos. Doses comparable to those of conventional polymeric microneedles were used. The needles remain embedded inside tissues and then undergo gradual degradation allowing for sustained release of the drug cargos [134]. Although developed for cutaneous melanoma, such devices present huge potential to treat ocular melanoma since the eye is easily accessible for materials implantation. 

A model of choroidal melanoma was used to evaluate ^125^I brachytherapy dose distribution in the presence of gold nanoparticles. The authors concluded that when loading the choroidal tumour volume with gold nanoparticles, the dose distribution improves by increasing the dose to the tumour and decreasing it to the healthy tissues [135]. Moreover, regarding brachytherapy improvement, a new eye plaque was experimentally validated for clinical use in larger ocular melanoma tumours. Physical and dosimetric measurements for the 24 mm plaque agreed with nominal specifications and theoretical predictions. In addition, this plaque provided greater basal coverage and lower surface doses than existing plaques [136]. 

## 4. Final Considerations

This review presents the currently available treatments for melanocytic neoplasms. An early diagnosis and a detailed case evaluation are essential for treatment effectiveness. The diagnosis should be guided by detailed ophthalmological exams and supported by anatomopathological evaluation. Understanding these neoplasms’ cell biology and the incidence is necessary to perform a correct diagnosis and achieve the best prognosis. The treatment options should be carefully chosen, considering the patient’s, lesions, and owners features. Importantly, continuous monitoring of the animal is fundamental to control tumour recurrences or metastases. New therapies in onco-ophthalmology are being developed, from bioengineered vaccines to biomaterials and drug delivery systems. These possibilities might offer patients vision preservation and provide well-being and quality of life in the near future. 

## Data Availability

Data are contained within the article.
